# Effect of Wearing Surgical Face Masks on Gas Detection from Respiration Using Photoacoustic Spectroscopy

**DOI:** 10.3390/molecules27113618

**Published:** 2022-06-04

**Authors:** Cristina Popa, Mioara Petrus, Ana Maria Bratu

**Affiliations:** Laser Department, National Institute for Laser, Plasma and Radiation Physics, 409 Atomistilor St., P.O. Box MG-36, 077125 Magurele, Romania; cristina.achim@inflpr.ro

**Keywords:** surgical face masks, ethylene, carbon dioxide, photoacoustic spectroscopy method

## Abstract

Wearing surgical face masks is among the measures taken to mitigate coronavirus disease (COVID-19) transmission and deaths. Lately, concern was expressed about the possibility that gases from respiration could build up in the mask over time, causing medical issues related to the respiratory system. In this research study, the carbon dioxide concentration and ethylene in the breathing zone were measured before and immediately after wearing surgical face masks using the photoacoustic spectroscopy method. From the determinations of this study, the C_2_H_4_ was established to be increased by 1.5% after one hour of wearing the surgical face mask, while CO_2_ was established to be at a higher concentration of 1.2% after one hour of wearing the surgical face mask, when the values were correlated with the baseline (control).

## 1. Introduction

The widespread use of face masks reduces the spread of SARS-CoV-2 by minimizing the excretion of respiratory droplets from asymptomatic contaminated individuals or individuals who have not yet manifested symptoms [1,2]. This advice became disputed and even politicized in some places, because of concerns about the safety of masks [1,2,3]. Some studies [3,4,5,6,7,8,9,10] raised concerns related to hypercapnia (a condition arising from too much carbon dioxide in the blood) and hypoxemia (a condition arising from too low oxygen in the blood than normal) caused by wearing face masks.

Guidance about mask wearing has varied from country to country, and some health bodies, including the World Health Organization, have modified their advice over time [1,2,11]. Some research has been conducted so far in this field: a study conducted by Otmar Geiss in 2020 [11] reported CO_2_ concentrations ranging from 2150 to 2875 ppm and no differences were observed among the types of face masks tested. According to the literature, these concentrations have no toxicological effect [1,6,11]. However, concentrations in the detected range can cause undesirable symptoms, such as fatigue, headache, and loss of concentration.

Another study [12] (Dattel et al., 2020) explored the effects of face masks on CO_2_, heart rate, respiration rate, and oxygen saturation on instructor pilots with relatively high CO_2_ concentrations (around 45,000 ppm) detected. Michelle S. M. Rhee et al., 2021 [13], reported that CO_2_ increases with face masks but remains below short-term National Institute for Occupational Safety and Health limits.

The research of Li et al. (2005) [14] investigated the effects of wearing face masks, with and without nano-functional treatments, on thermo-physiological responses and the subjective perception of discomfort in five healthy participants; they found that surgical face masks were rated significantly lower for perceptions of humidity, heat, breath resistance, and overall discomfort, than N95 face masks. In all these studies, found in the literature [1,2,3,4,5,6,7,8,9,10,11,12,13,14], ethylene from respiration was not among the investigated parameters.

In plants, ethylene is a major plant hormone mediating developmental processes and stress responses to stimuli, such as infection [15,16]. In humans, the elevation of breath levels of ethylene is associated with a variety of metabolic and pathologic conditions [17] because it is produced during systemic inflammation and is released in exhaled breath. Systemic oxidative stress correlates ethylene as a terminal product of the oxidation of omega-3 PUFA. Practically, ethylene is a by-product of lipid peroxidation and can be used to assess free radical damage.

The existing breath detection techniques designed to analyze air samples containing various volatile organic compounds include GC-MS/PTR-MS techniques, electronic nose (e-nose) [18] and infrared spectroscopy.

Compared with non-spectral procedures, such as catalytic combustion, semiconductor gas sensing and electrochemical methods, there is no chemical reaction and no consumption of detection materials in the photoacoustic spectroscopy gas detection process, which makes the safety, stability, and life of a gas detection system highly improved [19]. Compared with other spectral methods, photoacoustic spectroscopy gas detection technology is not used to directly measure the change of light intensity, but to detect the acoustic signal produced by photoacoustic effect [20]. Therefore, it is an indirect measurement method without background signal interference. Because of these advantages, photoacoustic spectroscopy gas detection technology is widely used in medical, industrial production [21,22,23], environmental, [24,25,26], monitoring, and other fields [27,28,29].

This study aimed to determine the concentration of ethylene and carbon dioxide from human respiration before and after one hour of wearing surgical face masks using photoacoustic spectroscopy gas detection technology.

## 2. Results

This research assesses the effect of wearing surgical face masks using laser photoacoustic spectroscopy gas detection in the evaluation of ethylene and carbon dioxide.

Taking into account that the number of the absorbing molecules is proportional to the amplitude of the signal, ethylene and carbon dioxide emission was established in the case of 4 healthy volunteers aged between 31 and 41 years. 

**Figure 1:**
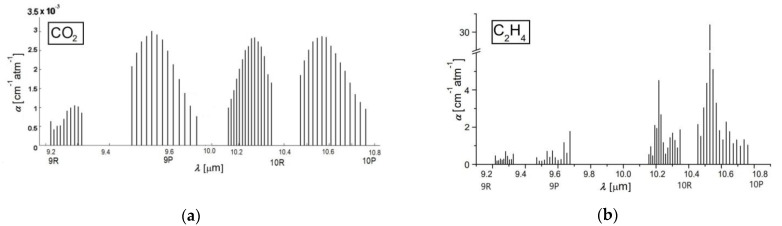
The absorption coefficients at laser lines of (**a**) carbon dioxide and (**b**) ethylene [32,33].

A chemical compound that retains laser radiation is stimulated to a higher quantum condition causing a reduction in laser light intensity, which can be directly quantified via absorption spectroscopy [30,31,32]. The absorption characteristics distinctive to each chemical compound make it feasible to detect trace gases and establish their accumulations. Absorption factors (see Figure 1) are, in most cases, in the order of 1 cm^−1^. To improve the quantification of ethylene and carbon dioxide absorption coefficients, an adjusted process was adopted.

To determine gas absorptions, it is imperative to calibrate the resonant cell with a well-known gas compound and to establish the linearity of the detector with the concentration of the known gas over orders of magnitude. The linear responses of the cavity for the low detection limit of carbon dioxide and ethylene in literature is reported [32,33]. For the carbon dioxide absorption coefficients determination (see Figure 1b), we used a certified mixture containing 991 ppmV carbon dioxide in pure nitrogen certified and supplied by Linde Gas. We examined this mixture at a total pressure of approximately 1030 mbar and a temperature T ≅ 23 °C, using the commonly accepted value of the absorption coefficient of 3.01 × 10^−3^ cm^−1^atm^−1^ at 9P(18) laser transition.

In the case of the ethylene absorption coefficients determination (see Figure 1a) at the laser lines, we used a commercially prepared, certified mixture containing 0.96 ppm ethylene in pure nitrogen certified and supplied by Linde Gas. We examined this mixture at a total pressure of approximately 1030 mbar and a temperature T ≅ 23 °C using the commonly accepted value of the absorption coefficient of 30.4 cm^−1^atm^−1^ at the 10P(14) line of the laser.

All the measurements were made at the resonant cell pressure close to the atmospheric one and room temperature. So long as the absorption factors of carbon dioxide and ethylene at the separate laser wavelengths were specifically estimated [32,33], the CO_2_ laser was set at specific lines; first on the 9P (18) line: 9.53 μm where carbon dioxide showed a powerful assimilation, equivalent to an absorption coefficient of 3.01 × 10^−3^ cm^−1^atm^−1^ and then on the 10P (14) line 10.53 μm, where ethylene showed a powerful assimilation, equivalent to an absorption coefficient of 30.4 cm^−1^atm^−1^.

**Figure 2:**
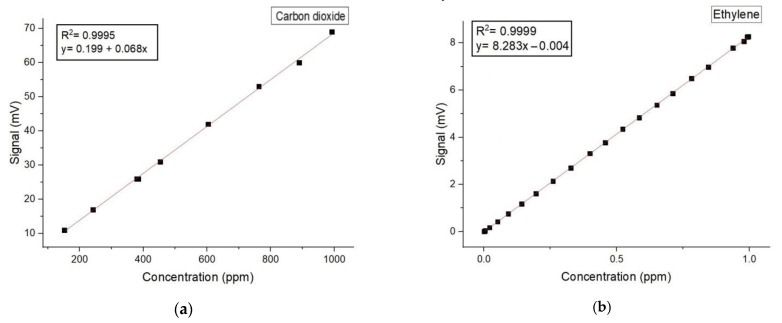
The concentration-dependent response for (**a**) carbon dioxide and (**b**) ethylene.

Below, in Figure 2, we can find the regression curve and the Pearson correlation coefficient for both carbon dioxide and ethylene.

Table 1 shows the parameters that were available by this application for the assessment of the gas molecules after wearing surgical face masks using laser photoacoustic spectroscopy gas detection in the evaluation of carbon dioxide and ethylene.

For the carbon dioxide and ethylene detection, we use software that records on different panels both the laser power, photoacoustic signal, and trace gases concentration.

A total of 4 participants (4 females) were included in the study. The age ranged from 31 to 41 years old with 35 ± 7.6 yr, BMI 22.52 ± 4.2 kg/m^2^. The participants were well-informed about the purpose and demands of the experimental study before giving their written consent to participate in this research. Information was asked regarding age, body weight and body height, time, and nature of the last meal and drink, recent exercise activity, medication, and smoking status.

The breath was analyzed for a single subject with 4 samples per day for a period of 2 months. The participants were non-smokers, non-alcoholics, non-renal, non-diabetic, and without chronic mental or physical health problems, and without any recent antibiotic therapy. Prior to the analysis of the breath, the participants were asked to avoid the following for at least 6 h before, or at any time during, the breath sample collection: alcohol and coffee, food or beverages, and to refrain from exercise in the morning. On the day prior to the test, products such as onions, leeks, eggs, and garlic should be avoided.

**Figure 3:**
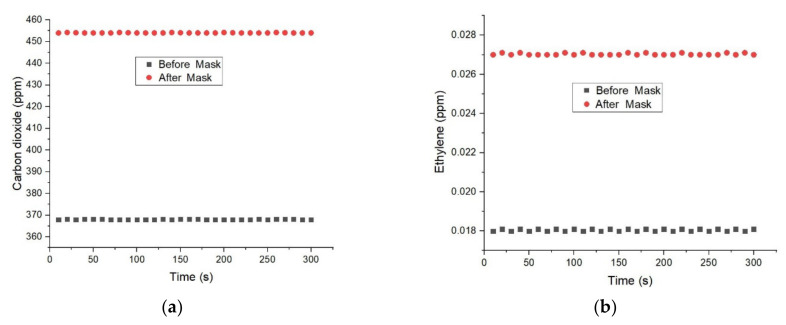
(**a**) Concentrations of CO_2_ measured before and after one hour of for surgical face mask wearing (**b**) Concentrations of ethylene measured before and after one hour of for surgical face mask wearing.

Figure 3 presents the concentration of carbon dioxide and ethylene from human respiration before and after one hour of wearing surgical face masks using photoacoustic spectroscopy gas detection technology.

The figure above shows the concentrations of carbon dioxide and ethylene measured for one type of face mask and breathing through the mouth in the breathing sampling kit. The results recorded for CO_2_ levels (see Figure 3a) found in the respiration before the wearing of a surgical face mask presented a corresponding CO_2_ concentration of 368 ppm. In the case of CO_2_ from human respiration after one hour of wearing the surgical face mask, we registered an increase in the photoacoustic signal with an equivalent carbon dioxide concentration of about 454 ppm.

When analyzing Figure 3b, the ethylene concentrations before the wearing of a surgical face mask presented a corresponding concentration of 0.018 ppm. In the case of ethylene from human respiration after one hour of wearing the surgical face mask, we registered an increase in the photoacoustic signal with an equivalent concentration of about 0.027 ppm.

To assess the reproducibility of the laser photoacoustic spectroscopy method the respiration was analyzed for both carbon dioxide and ethylene concentrations with four replicates per day before and after wearing the mask on three consecutive days (see Table 2 and Table 3).

The results for the carbon dioxide (Table 2) on individual days (intra-day) ranged from 365 ppm to 370 ppm (before wearing the face mask) with relative standard deviations (RSDs) from 0.7% to 0.96%, whereas, after one hour of wearing the face mask, the carbon dioxide from respiration was in the range of 452 ppm to 457 ppm with RSDs ranging from 0.5% to 0.64%. The overall (inter-day) results for the carbon dioxide before wearing the face mask were 368 ppm (RSD: 0.79%), and after wearing the face mask, 454 ppm (RSD: 0.6%), respectively. The results for the ethylene concentrations before wearing the face mask ranged from 0.017 ppm to 0.019 ppm with RSDs of 5.26% to 11.12%, and after one hour of wearing the face mask, ethylene values ranging from 0.023 ppm to 0.029 ppm with RSDs of 7% to13.1% were obtained. The inter-day results for ethylene before wearing the face mask were 0.018 ppm (RSD: 7.4%) and after wearing the face mask 0.027 mg/L (RSD 10.3%) (see Table 3). From the obtained results, the CO_2_ was established to be at a 1.2% higher concentration after one hour of wearing the surgical face mask, while ethylene was established to be increased by 1.5% after one hour of wearing the surgical face mask, when the values were correlated to the baseline (control). 

## 3. Discussion

A number of papers [11,12,13,14,34] have shown that CO_2_ increases with face masks when compared to controls. Otmar Geiss [11,34] reported a CO_2_ concentration increase of 725 ppm, whereas Dattel et al. [12,33], detected a CO_2_ concentration around 45,000 ppm. Similar to Otmar Geiss [11,33] and Dattel et al. [12,34], Michelle S. M. Rhee et al., 2021 [13,34] reported that CO_2_ increases with face masks but remains below short-term National Institute for Occupational Safety and Health limits.

Inhaled CO_2_ at lower concentrations (<10,000 ppm) has no toxicological effects while at higher concentrations (>50,000 ppm) it causes the development of hypercapnia and respiratory acidosis (Permentier et al., 2017; Otmar Geiss, 2020) [11,35]. A concentration of 5000 ppm is the workplace exposure limit (as 8 h TWA) in most jurisdictions. Exposures to increased inhaled CO_2_ concentrations between 2 and 3% (20,000–30,000 ppm) are known to produce sweating, headache and dyspnea (Schneider and Truesdell, 1922; Otmar Geiss 2020) [11,36]. Inhaled concentrations between 4 and 5% (40,000–50,000 ppm) are associated with dyspnea, increased blood pressure, dizziness, and headache (Schneider and Truesdale 1922; Schulte 1964; Otmar Geiss 2020) [11,36,37]. If inhaled CO_2_ concentrations are at 5% (50,000 ppm), mental depression may occur within several hours (Schulte, 1964; Otmar Geiss 2020) [36,37]. The CO_2_ concentrations measured in this study are all far below these threshold values and range between 368 ppm (before wearing a surgical mask) and 454 ppm (immediately after wearing a surgical mask).

Carbon dioxide-related health-symptoms have been observed at concentrations above 1000 ppm and these include drowsiness and loss of attention (Guais et al., 2011) [38]. A portion of the human population has been described as being sensitive to fluctuating CO_2_ concentrations. As a vasodilator, the effect on people prone to headache has also been discussed. For example, Lim et al. 2006 [39] administered a survey to healthcare workers to determine risk factors associated with the development of headaches. Approximately 40% of the respondents reported wearing face masks was associated with headaches. This study did not, however, report the inhaled CO_2_ concentrations. Satish et al. 2012 [40] suggested in their study that even moderately elevated CO_2_ concentrations (approximately 2500 ppm) have the potential to affect decision-making.

In all these studies found in the literature [1,2,11,12,13,14,34,35,36,37,38,39,40], ethylene from the human respiration of the participants wearing surgical face masks was not among the investigated parameters. The originality of our research is also due to the assessment of ethylene from healthy participant’s respiration, after wearing a surgical mask, using a modified photoacoustic spectroscopy gas detection system.

The ethylene concentrations measured in this study, similar to the carbon dioxide concentrations, are all far below the threshold values and range between 0.019 ppm (before wearing a surgical mask) and 0.023 ppm (immediately after wearing a surgical mask), with a 0.004 ppm increase after one hour of wearing a surgical face mask.

Face masks are essential components of personal protective equipment for health care workers in hospitals and public civilians alike. Notably, masks are helpful in preventing illness in healthy persons and preventing asymptomatic transmission, especially in a global pandemic. Although their use is now mandatory in many states due to COVID-19, they were generally used in hospitals and operating rooms even before the current pandemic. They do, however, increase both the carbon dioxide and ethylene concentrations behind them, as evidenced by this study. Although the results show that gases concentrations from the participant’s respiration increases after wearing a surgical mask for an hour, they remain below short-term National Institute for Occupational Safety and Health limits [1,2,11,12,13,14,34,35,36,37,38,39,40].

## 4. Materials and Methods

The research was devoted to verifying the possibility that gases from respiration could build up in the mask over time, causing medical issues related to the respiratory system.

So, the study evaluated the carbon dioxide and ethylene gases found in human respiration before and after wearing a surgical face mask using the CO_2_ laser photoacoustic spectroscopy method with respect to CO_2_ laser frequencies [15,16,17,22,30,31,32,41,42,43].

We used a specially designed sampling kit to collect a clean breath air sample with “alveolar” breath, which comes from the lungs, where gaseous exchange between the blood and breath air takes place.

The exhaled air is a heterogeneous gas, and for a healthy individual, the first part of an exhaled breath, roughly 150 mL, consists of “dead-space” air from the upper airways (such as the mouth and trachea), where air does not come into contact with the alveoli of the lungs. The following part of a breath, about 350 mL, is “alveolar” breath, which comes from the lungs, where gaseous exchange between the blood and breath air takes place. For volatile organic compounds (VOCs) exchanged between the blood and alveolar air, the dead-space air is a “contaminant”, diluting the concentrations of VOCs when breath air is collected.

For the identification of carbon dioxide and ethylene presence, all the participants’ respiration samples were collected in 0.75 L bags coated with aluminum and designed to accumulate and keep (for a maximum of 6 h) multiple respirations [44]. The participant positioned the piece in their mouth and naturally exhaled through the mouth into the breathing sampling kit. When a proper respiration is collected, the participant stops the natural exhalation. The bag with the collected breath is delivered to the laboratory and transferred into the resonant measuring cell where we can detect the traces of gases by the gas flow controller (MKS 1179A). The transfer of the participant’s sample gas respiration from the aluminum bag to the resonant cell was achieved at a controlled flow rate of 600 sccm, and the pressure of gases entered in the resonant cell was established with a Baratron pressure gauge. This way, the time required for the sample gas is approximately ~1.25 min for a flow rate of 600 sccm, and the final pressure inside the cell, measured for breath samples from the participants, is usually at ~800 mbar with an equivalent responsivity of 240 cmV/W.

**Figure 4:**
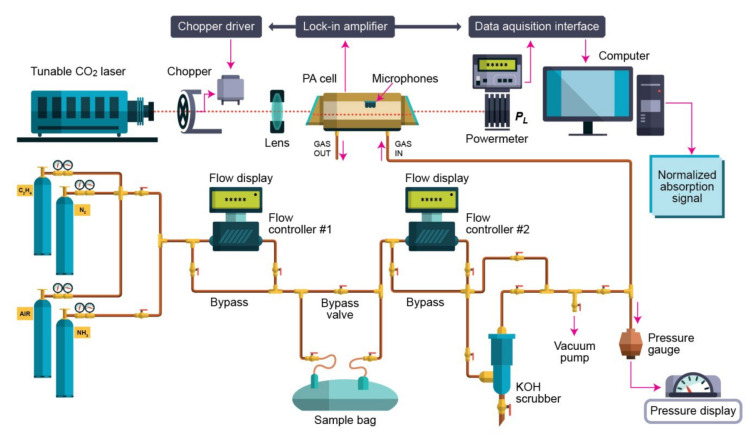
Spectroscopic set-up used for the evaluation of carbon dioxide and ethylene from the respiration of people wearing a surgical mask.

The photoacoustic spectroscopy gas detection used for the evaluation of the carbon dioxide and ethylene is summarized in Figure 4 and described in detail in [15,16,17,22,30,31,32,41,42,43,44].

The photoacoustic spectroscopy gas detection arrangement consists of a CO_2_ laser, a lens, a chopper, a photoacoustic resonant cell, a powermeter, a lock-in amplifier, an acquisition panel, and a data-processing computer. The experimental detection system also includes a gas handling system with an essential role in the control of the studied gases, but it can also perform other actions described in detail in other articles [41,42,43,44]. The photoacoustic cell is an indispensable module in photoacoustic spectroscopy gas detection technology because the whole photoacoustic effect occurs in the photoacoustic cell. The photoacoustic signal is inverse proportional to the cell volume, which requires the cell volume to be as small as possible. However, too small a cell volume will make the cell wall produce a background signal that cannot be eliminated, so it is very important to grasp the cell volume. Generally, a resonant photoacoustic cell can increase the photoacoustic signal through resonance, so the limit of detection (LOD) of a resonant photoacoustic cell is lower than that of a non-resonant cell, which is the reason why the resonant cell is paid more and more attention [32,44].

Basically, the CO_2_ laser photoacoustic spectroscopy gas detection system uses an adjustable laser and an H-type resonant cylindrical cell where the gas is detected. The laser beam enters the cylindrical cell after modulating and focusing. Similarly, to the photoacoustic cell, the microphone plays an important role in photoacoustic spectroscopy gas detection, and how to improve the sensitivity of the microphone has always been a hot topic.

In the experiments [15,16,17,22,30,31,32,41,42,43,44], the sample is enclosed in a resonant cell, where the acoustic waves are detected by four Knowles electret EK-3033 miniature microphones connected in series and mounted flush with the wall. Each microphone has a sensitivity of 20 mV/Pa and a total sensitivity of 80 mV/Pa. They are positioned at the loops of the standing wave pattern, at an angle of 90° to one another. The microphones are fixed to the resonator by holes with a 1 mm diameter, positioned on the central perimeter of the resonator. The battery-powered microphones are mounted in a Teflon ring pulled over the resonator tube. The signal is fed into a lock-in amplifier that provides the amplitude and phase of the photoacoustic signal. The value of the acoustic signal determined by the microphones and normalized to the size of the CO_2_ laser radiation power is comparable to the molecular absorption coefficient of the analyzed gas sample at a CO_2_-used laser radiation wavelength. The microphones turn the acoustic signal into an electrical signal that is detected by a lock-in amplifier with a role in setting the chopper frequency and providing the photoacoustic signal. After passage through the stainless steel and Teflon cylindrical cell, the power of the laser beam is measured by a laser radiometer. Its digital output is introduced in the data acquisition interface module together with the output from the lock-in amplifier. The signals are gathered by an acquisition card and the data are recorded by a computer interface. The number of the absorbing molecules from the cylindrical cell is proportional with the amplitude of the photoacoustic signal. All experimental data are processed and stored by a computer. The frequency stabilized, line tunable CO_2_ laser emits in the 9.2–10.8 µm range, where a large number of gas molecules possess a high absorption coefficient. The light beam was modulated with a high quality, low vibration noise, a variable speed between 4 and 4000 Hz, and a mechanical chopper with a 30-slot aperture, operated at the appropriate resonant frequency of the H-type resonant cylindrical cell (564 Hz). The laser beam diameter is typically 5 mm at the point of insertion of the chopper blade and is nearly equal to the width of the chopper aperture. An approximately square waveform was produced with a modulation depth of 100% and a duty cycle of 50% so that the average power measured by the powermeter at the exit of the photoacoustic detector was half the cw value. The diverging infrared laser beam is converged by a ZnSe focusing lens (*f* = 400 mm). In this way, a slightly focused laser beam is passed through the resonant cylindrical cell without wall interactions. The useful data are automatically recorded, in real time, via a Test Point acquisition card under computer control.

The dedicated program employed to acquire the data and calculate the value of the gas concentration was implemented using the Test Point platform. Further characteristics about the experimental set-up can be noted in the papers previously published [15,16,17,22,30,31,32,41,42,43,44].

## 5. Conclusions

Careful investigations of the carbon dioxide and ethylene concentrations of participants wearing surgical masks were performed using the CO_2_ laser photoacoustic spectroscopy gas detection method.

Our study demonstrates a non-significant increase in end tidal carbon dioxide and ethylene concentrations among participants while donning a surgical mask for one hour. According to the literature, these concentrations have no toxicological effect when inhaled. Therefore, there should not be concern for their regular day-to-day use. However, concentrations between 1000 ppm and 10,000 ppm can cause undesirable symptoms, such as fatigue, headache, and loss of concentration. This may be relevant for those segments of the population who are required to wear face masks over prolonged periods of time, such as students, bus drivers or cashiers, as well as persons with respiratory diseases. Although we recommend widespread mask usage, particularly during a respiratory disease pandemic, carbon dioxide and ethylene toxicity might complicate its use. Citizens should be provided regular opportunities to take breaks from the mask. A system to report symptoms related to mask use will be beneficial. A potential remedy is to change the surgical mask more often or designate an area to remove masks temporarily to mitigate any physiological effects associated with its use.

In summary, the current work was carried out by implementing a methodology that assured better conditions to measure real concentrations of carbon dioxide and ethylene from human respiration. 

The CO_2_ laser photoacoustic spectroscopy gas detection method can be a new and easier method to assess molecules, which may contribute to a better understanding of the effect of wearing surgical face masks on gas detection from respiration. The implication of elevated carbon dioxide and ethylene levels with the long-term use of surgical masks needs further studies.

## Figures and Tables

**Table 1:** Measurable factors for gases detection in human respiration after wearing surgical face masks.

Parameter	Value
Aluminum coated bags kit	0.75 L
Resonant cell frequency	564 Hz
Resonant cell pressure	≈1030 mb
Resonant cell volume	1000 cm^3^
Total microphone sensitivity	80 mV/Pa
Gas absorption coefficient	carbon dioxide- 9P(18); λ = 9.53 μm; α = 3.01 × 10^−3^ cm^−1^atm^−1^ ethylene-10P(14); λ = 10.53 μm; α = 30.4 cm^−1^atm^−1^
Temperature	≈23–25 °C
Responsivity	240 cmV/W
Human respiration time analysis	≈300 s
Nitrogen air outflow composition	Linde gas: nitrogen 5.0 (purity 99.999%) and 6.0 (purity 99.9999%)
Ethylene composition	Linde gas: 0.96 ppmV (±5%) C_2_H_4_ diluted in nitrogen 5.0 (purity 99.999%)
Carbon dioxide composition	Linde gas: 991 ppm in pure nitrogen
Carbon dioxide retention	KOH pellets
Water retention	Silica gel pellets
Q — quality factor of the system	16.1
CO_2_ laser operating mode	TEM_00_
Flow rate	600 sccm

**Table 2:** Intra- and inter-day repeatability: carbon dioxide measurements.

CO_2_ Experiments	Replicates before Wearing the mask	Average before Wearing the Mask (ppm)	SD before Wearing the Mask (ppm)	Rel. SD %	Replicates after Wearing the Mask	Average after Wearing the Mask (ppm)	SD after Wearing the Mask (ppm)	Rel. SD %
**day 1**	4	370	2.6	0.7	4	452	2.9	0.64
**day 2**	4	365	3.51	0.96	4	457	2.3	0.5
**day 3**	4	370	2.6	0.7	4	453	2.8	0.62
**inter-day**	12	368	2.89	0.79	12	454	2.7	0.6

**Table 3:** Intra- and inter-day repeatability: ethylene measurements.

CO_2_ Experiments	Replicates before Wearing the mask	Average before Wearing the Mask (ppm)	SD before Wearing the Mask (ppm)	Rel. SD %	Replicates after Wearing the Mask	Average after Wearing the Mask (ppm)	SD after Wearing the Mask (ppm)	Rel. SD %
**day 1**	4	0.017	0.001	5.88	4	0.029	0.003	10.4
**day 2**	4	0.018	0.002	11.12	4	0.023	0.003	13.1
**day 3**	4	0.019	0.001	5.26	4	0.029	0.002	7
**inter-day**	12	0.018	0.0013	7.4	12	0.027	0.0028	10.3

## Data Availability

Not applicable.
